# Oral amoxicillin challenges for low-risk penicillin-allergic patients at a large Veterans Affairs facility: a retrospective feasibility analysis

**DOI:** 10.1017/ash.2023.532

**Published:** 2024-01-11

**Authors:** Jessica M. Guastadisegni, Misozi K. Kolala, Jazmyn M. Criss, Marcus A. Kouma, Donald F. Storey, Reuben J. Arasaratnam

**Affiliations:** 1 Veterans Affairs North Texas Health Care System, Dallas, TX, USA; 2 Veterans Affairs North Texas Health Care System and Dallas VA Research Corporation, Dallas, TX, USA; 3 Veterans Affairs North Texas Health Care System and University of Texas Southwestern Medical Center, Dallas, TX, USA

## Abstract

We retrospectively reviewed the records of 136 veterans with a penicillin allergy label during a quality improvement initiative. We identified 82 inpatients eligible for removal of penicillin allergy by oral amoxicillin challenge, including 40 out of 82 (48%) still eligible after accounting for other limiting factors.

## Introduction

Unverified penicillin allergy labels are common and associated with harmful consequences to both individuals and health systems.^1^ The United States Centers for Disease Control and Prevention and various national allergy, infectious disease, and pharmacy specialty societies endorse proactive penicillin allergy testing. A recent American Academy of Allergy, Asthma & Immunology position statement supports such evaluation, even during routine healthcare encounters that do not require antibiotic therapy.^2^

Verification of penicillin allergy has historically required an allergist assessment in the outpatient setting using penicillin skin testing (PST), with or without subsequent oral amoxicillin challenge. However, a national shortage of and lack of access to allergy specialists are major obstacles to widespread penicillin allergy evaluation.^3^ Though the expansion of penicillin allergy evaluation driven by pharmacists has been successful,^4^ there is still a great need for more accessible and equitable approaches to this problem.

A recent international, randomized controlled trial comparing direct oral amoxicillin challenge against PST (with or without subsequent oral amoxicillin challenge) in patients with low-risk penicillin allergies achieved similar success (99.5% vs 97.9%) in removing the allergy label and noninferiority in relation to the incidence of immune-mediated adverse events (0.5% vs 0.5%).^5^ Given the reduced time and fewer resources needed to perform oral amoxicillin challenges in relation to PST, this modality of penicillin allergy evaluation could enable widespread delabeling efforts by non-allergy personnel across a variety of healthcare settings.

We initiated a penicillin allergy evaluation program using direct oral amoxicillin challenge in 2022. To inform our implementation strategy, we conducted a retrospective feasibility analysis of potential barriers to removing veterans’ penicillin allergy labels using this approach in the inpatient setting.

## Methods

The Dallas VA Medical Center is a 1A critical access facility with 244 acute care inpatient beds. The medical service includes an infectious diseases section with four physician and three pharmacist Full-Time Equivalents but without any on-site allergy/immunology staff. We retrospectively reviewed individual records of veterans with a penicillin allergy label selected from two, three-month convenience samples of acute care admissions within calendar years 2021 and 2022. We included veterans with a documented allergy to any of the following medications: penicillin, amoxicillin, ampicillin, amoxicillin-clavulanate, or nafcillin. For veterans with a penicillin allergy label who were admitted more than once during the analysis period, only the first admission was counted. We collected data on patient demographics, admission diagnoses, penicillin allergy label details, comorbidities, and length of stay. Risk of true penicillin allergy was based on a stratification proposed by Shenoy and colleagues^6^ and modified for our population. No increased risk referred to prior index reaction with proven safe receipt of penicillin after; intolerance history–non-allergy symptoms (e.g., GI upset); low risk–self-limited rash/pruritis (at any point), unknown reaction in childhood or more than 10 years ago, urticaria only more than 10 years ago, family history; moderate-high risk–anaphylaxis or angioedema at any point or the following in the last 10 years: urticaria, bronchospasm, loss of consciousness, severe GI symptoms, or unknown reaction at unknown time documented within last 10 years; very high risk–severe cutaneous adverse reactions, delayed severe reactions (acute generalized exanthematous pustulosis, Stevens-Johnson syndrome, drug reaction with eosinophilia and systemic symptoms, and toxic epidermal necrolysis), serum sickness, acute interstitial nephritis, and drug-induced liver injury. Feasibility of performing an oral amoxicillin challenge to remove a penicillin allergy label was determined based upon a composite criterion of low-risk penicillin allergy, a minimum 2-day admission (a time frame determined by authors that would allow identification of patients, patient interview, and administration of oral amoxicillin challenge and observation), and the absence of the following comorbidities—altered mental status or cognitive impairment, inability to consent, severe cardiac or respiratory failure, suspected drug reaction at time of admission, rash, nausea/vomiting, abdominal pain or inability to take oral medications. Facility leadership reviewed this project and designated it as nonresearch. Thus, Institutional Review Board approval was waived.

## Results

Baseline demographic characteristics of 136 veterans with a penicillin allergy label are shown in Table 1. Most patients were male (89.7%) and white (61.0%), with the median age of 71 years (IQR, 63–76). The median Charlson Comorbidity Index was 5 (IQR, 3–7), driven primarily by high prevalence of diabetes mellitus (51.5%), solid tumors (29.4%), and congestive heart failure (27.9%). Nearly two-thirds of admissions were for noninfectious reasons.

**Table 1.** Baseline characteristics of veterans admitted with penicillin allergy label

Characteristics	*N* = 136
Age, median (IQR), y	71 (63–76)
Sex, No. (%)	
Female	14 (10.3%)
Male	122 (89.7%)
Race and ethnicity, No. (%)	
White	83 (61.0%)
Black or African American	41 (30.1%)
Other	12 (8.8%)
Charlson Comorbidity Index score, median (IQR)	5 (3–7)
Charlson Comorbidity Index components, No. (%)^a^	
Myocardial infarction	12 (8.8%)
Congestive heart failure	38 (27.9%)
Peripheral vascular disease	20 (14.7%)
Cerebrovascular accident or transient ischemic attack	24 (17.6%)
Hemiplegia	1 (0.7%)
COPD	33 (24.3%)
Diabetes without complications	39 (28.7%)
Diabetes with end organ damage	31 (22.8%)
Moderate or severe renal disease	7 (5.1%)
Mild liver disease	10 (7.4%)
Moderate to severe liver disease	3 (2.2%)
Peptic ulcer disease	5 (3.7%)
Localized solid tumor	36 (26.5%)
Metastatic solid tumor	4 (2.9%)
Leukemia	1 (0.7%)
Lymphoma	3 (2.2%)
Dementia	6 (4.4%)
Rheumatic or connective tissue disease	16 (11.8%)
HIV or AIDS	0 (0%)
Reason for admission, No. (%)	
Infection-related or treated with antibiotics	47 (34.6%)
Noninfection-related, no antibiotics received	89 (65.4%)
Type of penicillin allergy documented in EHR, No. (%)	
Penicillin	124 (91.2%)
Amoxicillin	3 (2.2%)
Amoxicillin-clavulanate	6 (4.4%)
Ampicillin	2 (1.5%)
Nafcillin	1 (0.7%)
Timing since index reaction, median (IQR), y	18 (8-23)
Observed allergic reaction, No. (%)	8 (5.9%)
Health professional who entered allergy, No. (%)	
MD	26 (19.1%)
PA/NP	9 (6.6%)
Pharmacist	40 (29.4%)
Nurse	42 (30.9%)
Other	19 (14.0%)
Reaction listed, No. (%)^a^	
Unknown	55 (40.4%)
Rash (not urticaria/hives)/pruritis	40 (29.4%)
Urticaria/hives	21 (15.4%)
Anaphylaxis or angioedema^b^	18 (13.2%)
SCAR or organ-specific injury	2 (1.5%)
GI side effect	8 (5.9%)
Localized nonfacial swelling	2 (1.5%)
Other	6 (4.4%)
Treatment given for reaction, No. (%)	
Yes	3 (2.2%)
No	13 (9.6%)
Unknown	120 (88.2%)
Concurrent other antibiotic allergies listed, median (IQR)	0 (0–0)
Concurrent nonantibiotic allergies listed, median (IQR)	1 (0–2)
Prescribed β-lactam after index reaction, No. (%)	
No	68 (50.0%)
Penicillin	11 (8.1%)
Cephalosporin	63 (46.3%)
Carbapenem	9 (6.6%)
Inpatient length of stay ≥2 weekdays, No. (%)	103 (75.7%)
Risk stratification, No. (%)^c^	
No increased risk	10 (7.4%)
Intolerance history	8 (5.9%)
Low risk	82 (60.3%)
Moderate-high risk	34 (25.0%)
Very high risk	2 (1.5%)
PEN-FAST score, median (IQR)^d^	1 (1–3)
Feasibility of performing oral amoxicillin challenge, No. (%)^e^	40 (48.8%)

Note. EHR, electronic health record; GI, gastrointestinal; PEN-FAST, Penicillin Allergy Decision Rule; SCAR, severe cutaneous adverse reaction.

a Categories are not mutually exclusive as patients may have had more than one comorbidity or symptom listed; percentages may add to more than 100%.

b Includes reactions documented as “swelling” and for which angioedema could not be excluded.

c Patients with confirmed safe receipt of any penicillin-class antibiotic other than piperacillin-tazobactam after the index date were classified as “No increased risk.” Those who received piperacillin-tazobactam were reclassified as “No increased risk” only if the original history was consistent with a “low-risk” allergy.

d PEN-FAST: PEN, penicillin allergy reported by patient; F, five years or less since reaction (two points); A, anaphylaxis or angioedema (two points); S, severe cutaneous adverse reaction (two points); T, treatment required for reaction (one point); 0 points: very low risk of positive penicillin allergy test <1%, 1–2 points: low risk 5%, three points: moderate risk 20%, 4–5 points: high risk 50%.^11^

e Percentage was calculated based on a denominator of *n* = 82 low-risk patients.

Of the penicillin-class of antibiotics listed as an allergy, penicillin was the most frequently listed (91.2%), followed by amoxicillin-clavulanate (4.4%), amoxicillin (2.2%), ampicillin (1.5%), and nafcillin (0.7%). The most frequently documented reaction was non-urticarial rash (29.4%), followed by urticaria/hives (15.4%), though a plurality (40.4%) of reactions were unknown. Based on careful review of the electronic health record (EHR), we determined 18 (13.2%) patients had allergy histories consistent with anaphylaxis or angioedema, and two patients (1.5%) had histories of severe cutaneous adverse reactions, organ-specific injury (interstitial nephritis), or serum sickness syndrome. Notably, the median time from the index reaction to admission (as determined from the EHR) was 18 years. Most of the allergy documentation in this cohort was historical (94%) and not directly observed.

Eighty-two patients (60.3%) were considered to have histories suggestive of low-risk penicillin allergy and so were eligible candidates for allergy label removal following direct oral amoxicillin challenge (Table 1). After accounting for insufficient duration of inpatient stay (*n* = 16) and precluding comorbidities (*n* = 26), approximately half (40/82) of these low-risk patients would have met our composite feasibility criterion for an amoxicillin challenge.

## Discussion

We conducted a retrospective feasibility analysis in our inpatient veteran population to determine the potential for penicillin allergy removal using direct oral amoxicillin challenge. We identified ample opportunities to remove penicillin allergy labels either via oral amoxicillin challenge or directly, via history and careful review of the EHR, lending support to the initiation of a pilot penicillin allergy removal program at our facility. Based on our results (a total of 100 inpatients over 6 months total with low-risk penicillin allergy, intolerance, or no increased risk history), we estimated approximately 10–15 patients per month could have their penicillin allergy label removed, requiring about 20–25 hours per month of combined physician/pharmacist/nursing time.

A short duration of inpatient stay or certain comorbidities would have reduced the feasibility of an amoxicillin challenge among our low-risk patients by approximately 50%. These findings highlight the need for thoughtful, context-specific implementation strategies to remove penicillin allergies in an elderly veteran population. Examples may include third-party consent to oral amoxicillin challenge for patients with cognitive impairment or systematized, close patient follow-up to reassess the feasibility of performing challenges should their acute admission-related diagnosis resolve.

Because the prevalence of penicillin allergy increases with age,^7^ it is important to increase delabeling efforts in older persons such as our hospitalized veteran cohort. In the US Drug Allergy Registry, it was recently reported that 286 out of a potential 296 adults aged 65 years or older were able to have their penicillin allergy removed.^8^ A review of published literature on penicillin allergy within the Veterans Health Administration revealed that allergist-provided or pharmacy-driven PST remained the most common modality of penicillin allergy evaluation.^8^ Although a voluntary pharmacy-driven penicillin allergy label removal program (including the use of oral amoxicillin challenges) has begun across the Veterans Health Administration,^9^ our findings suggest more detailed study is needed with regard to potential implementation barriers.

This study has some limitations. These data were collected to inform a quality improvement initiative specific to our facility and thus cannot be generalized across other facilities and non-VA populations. EHR review is unlikely to confer the same accuracy with respect to penicillin allergy history as in-person interviews and thus may have led to the misclassification of penicillin allergy risk. Furthermore, the retrospective nature of this report could not identify point-of-care barriers to delabeling such as provider and patient hesitancy.

In summary, we found ample opportunity to remove penicillin allergy labels with an oral amoxicillin challenge among inpatients at our facility. Careful consideration of other patient-specific and/or health systems variables may improve the feasibility of this approach among an elderly population of hospitalized veterans.
